# Supplementary material for: Cytoplasmic control of Rab family small GTPases through BAG6
Source: EMBO Rep. 2019 Feb 25;20(4):e46794. doi: 10.15252/embr.201846794 (PMC6446207; doi:10.15252/embr.201846794)
Supplement: Supplementary file 1 — Appendix [file EMBR-20-e46794-s001.pdf]

Appendix Supplementary Information

Cytoplasmic control of Rab family small GTPases through BAG6.

Takahashi *et al.*

Appendix table of contents

Appendix Figure S1. -- p. 2

Appendix Figure S2. -- p. 4

Appendix Figure S3. -- p. 6

Appendix Figure S4. -- p. 8

Appendix Figure S5. -- p. 10

Appendix Figure S6. -- p. 12

Appendix Figure S7. -- p. 14

Appendix Figure S8. -- p. 16

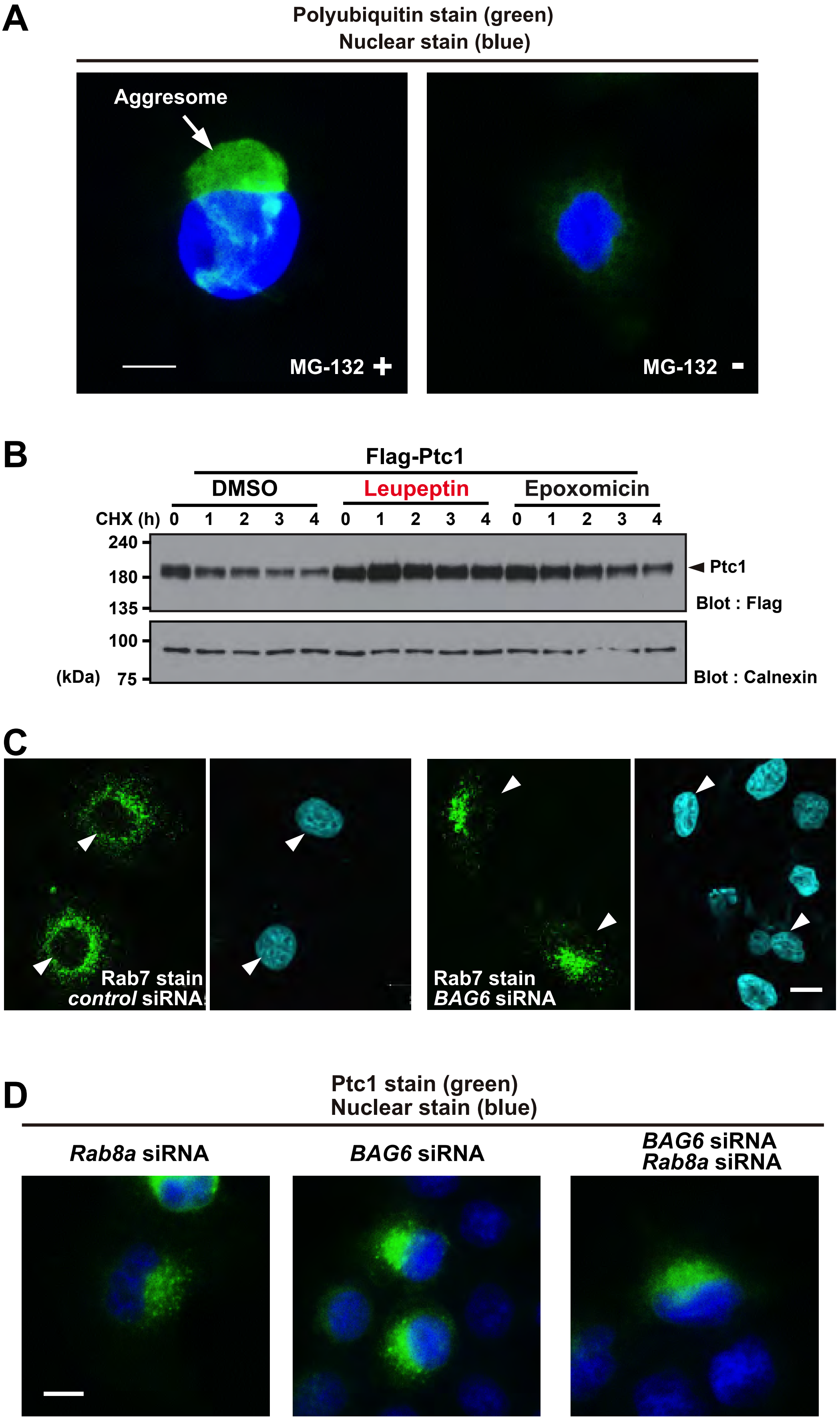

Appendix Figure S1, Takahashi et al

Appendix Figure S1.

The effects of protease inhibitors or BAG6 knockdown on endosomal proteins (related to Figs 1 and EV1).

(A) HeLa cells were treated with MG-132 (10 μM) for 12 h, and fixed cells were stained with anti-polyubiquitin FK2 antibody (green) under identical experimental condition to Fig EV1A. Aggresome, a perinuclear insoluble aggregate induced by MG-132-treatment, was stained by anti-polyubiquitin FK2 antibody (indicated by an arrow). The positions of the nucleus are shown by Hoechst staining (blue). Scale bar: 10 μm.

(B) Ptc1 is degraded in a lysosome-dependent manner. HeLa cells expressing Flag-tagged Ptc1 were treated with 20 μg/mL CHX and 0.1% DMSO (as a negative control), 10 μM leupeptin (as a lysosome inhibitor), or 10 μM epoxomicin (as a proteasome inhibitor) for the indicated periods. Calnexin was used as a loading control.

(C) Immunostaining of the endosomal marker protein Rab7 (green) in HeLa cells that were treated with (+) or without (-) *BAG6* siRNA. Nuclei are indicated by arrowheads. Scale bar: 10 μm.

(D) Immunostaining of the Ptc1 (green) in HeLa cells that were treated with *Rab8a* siRNA (left), *BAG6* siRNA (center) and their combination (right). The positions of the nucleus are shown by Hoechst staining (blue). See also Fig 1B and Fig EV1B. Scale bars: 10 μm.

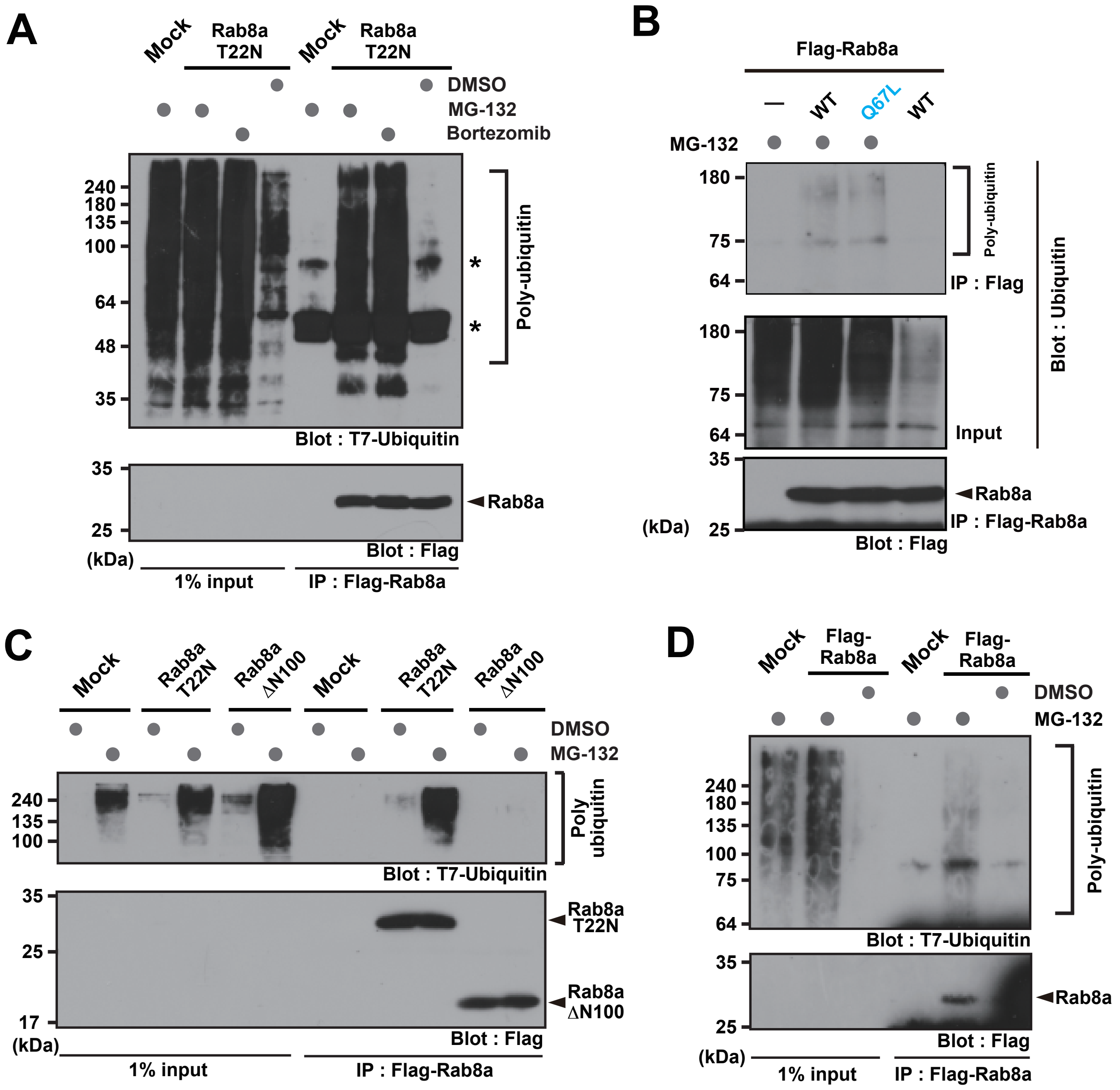

Appendix Figure S2, Takahashi et al

Appendix Figure S2.

Rab8a (T22N) is covalently polyubiquitinated (related to Figs 3H and 4E).

(A) Treatment with the proteasome inhibitors bortezomib and MG-132 stimulated the co-precipitation of polyubiquitin chains with Rab8a (T22N). The Flag-tagged Rab8a mutant and T7-tagged ubiquitin were co-expressed in HeLa cells and the cells were treated with (+) or without (-) 10 μM MG-132. After 4 h, Flag-precipitates were blotted with anti-T7 and anti-Flag antibodies. Asterisks indicate non-specific bands.

(B) Highly stable Rab8a (Q67L) was subjected to polyubiquitin modification only at a low level even in the presence of the proteasome inhibitor (see also Fig 3H).

(C) Polyubiquitin modification was not observed in N-terminally truncated Rab8a (ΔN100), in contrast to the case of full-length Rab8a (T22N) protein.

(D) Rab8a (T22N) is covalently modified with ubiquitin chains. Flag-tagged Rab8a and T7-tagged ubiquitin were expressed with (+) or without (-) 10 μM MG-132. Proteins in whole cell lysates were denatured by 1% SDS, diluted the denatured lysates in an IP buffer, and then Rab8a (T22N) protein was immunoprecipitated with an anti-Flag antibody. This denaturing procedure did not abolish polyubiquitin co-immunoprecipitation with Rab8a (T22N).

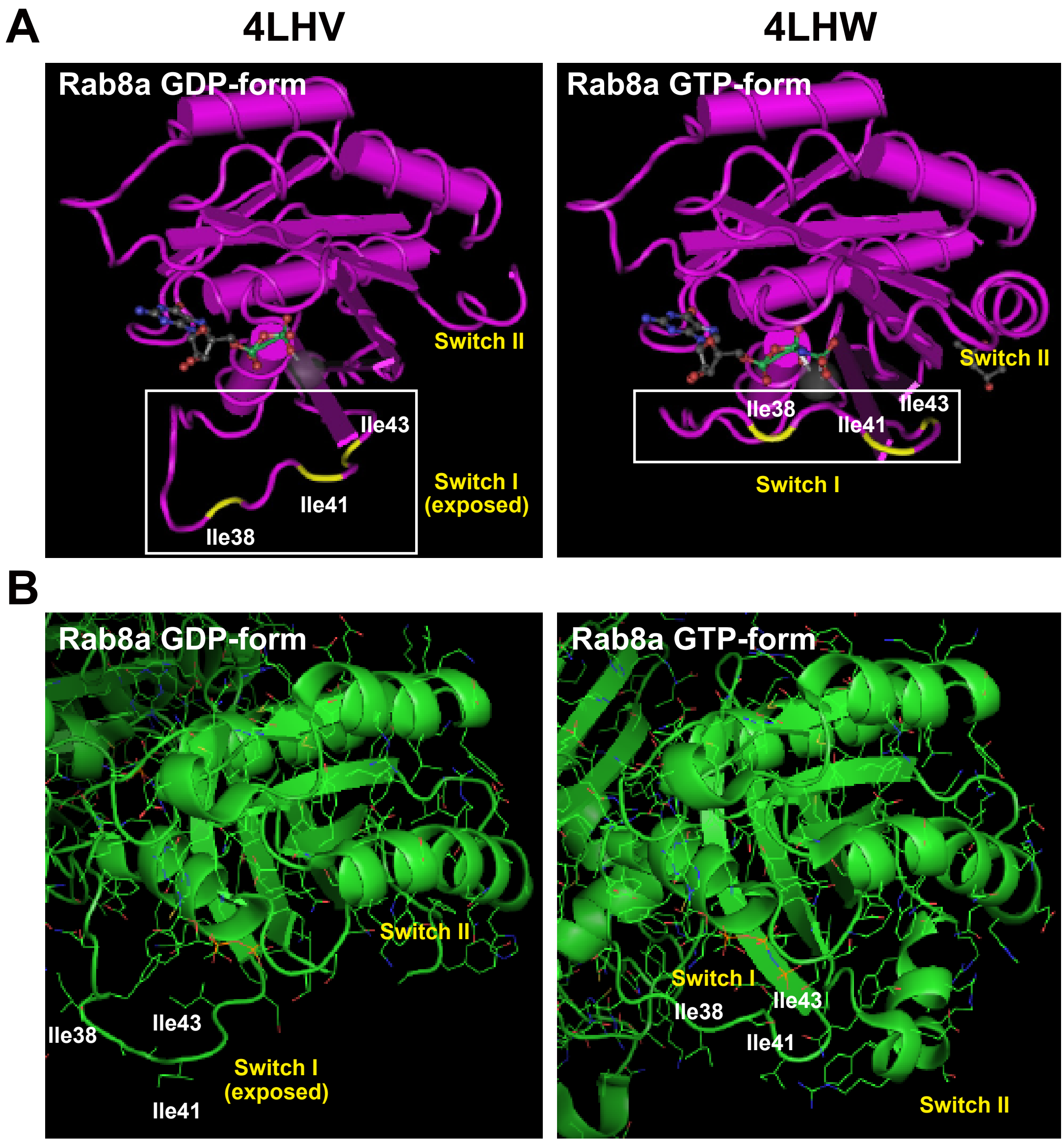

Appendix Figure S3, Takahashi et al

Appendix Figure S3.

Hydrophobic residues in the Rab8a Switch I region are exposed in its GDP bound form (related to Fig 4).

Conformations of the Rab8a GTPase domain in the GDP-bound (4LHV) and GTP-bound (4LHW) forms, respectively [16]. Two Switch regions of Rab8a, namely, Switch I (white box) and Switch II, are indicated (A). Note that the hydrophobic side chains of Ile^{38}, Ile^{41}, and Ile^{43} residues are exposed in its GDP-bound form (B).

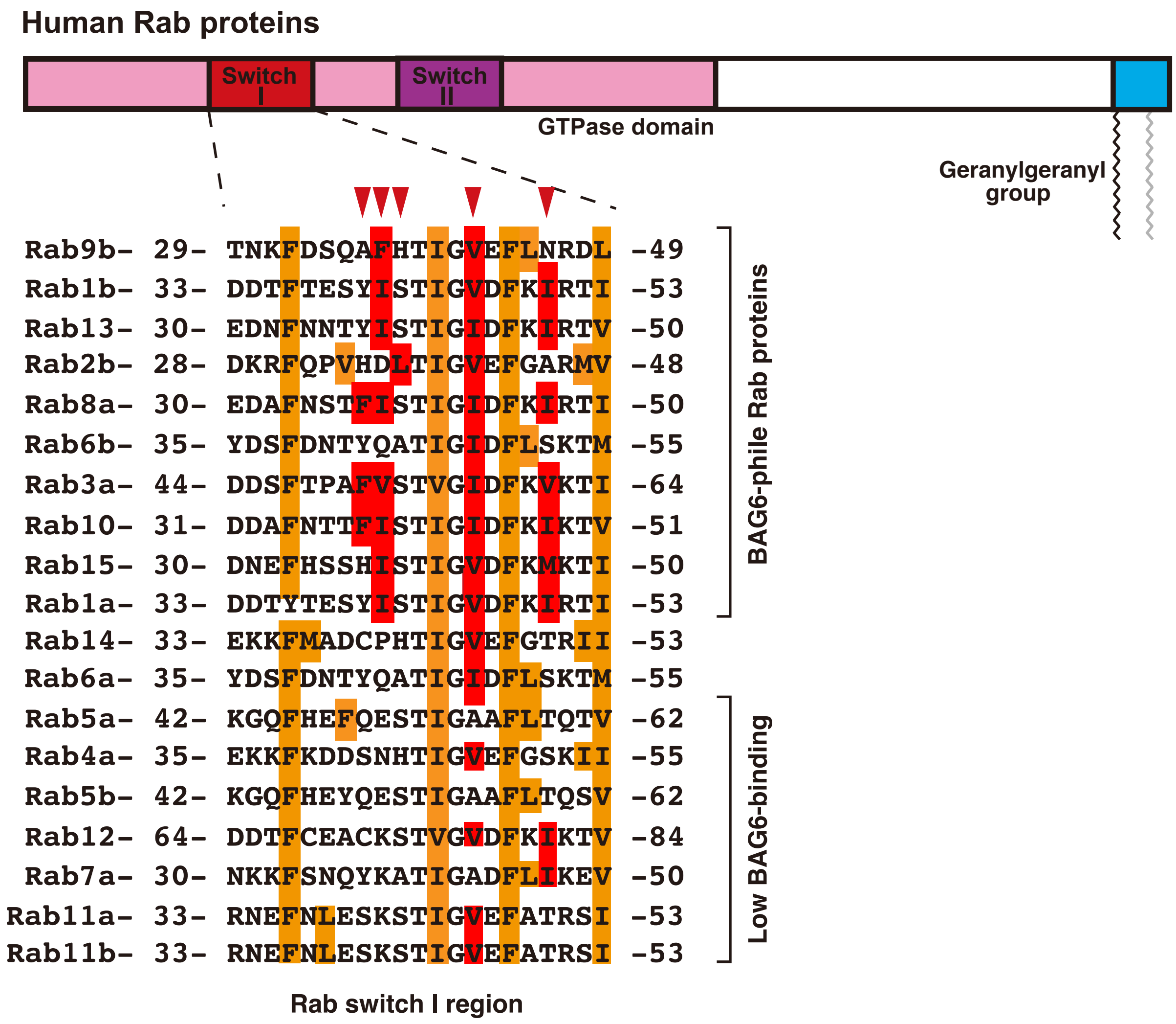

Appendix Figure S4, Takahashi et al

Appendix Figure S4.

Amino acid sequence alignments of the Switch I region of the Rab proteins (related to Figs 4A and 6B).

The distribution of the hydrophobic residues in the Switch I region is indicated in color. The hydrophobic residues that are frequently found in BAG6-phile Rab proteins are shown in red (indicated by red arrowheads), while the other hydrophobic residues are shown in orange.

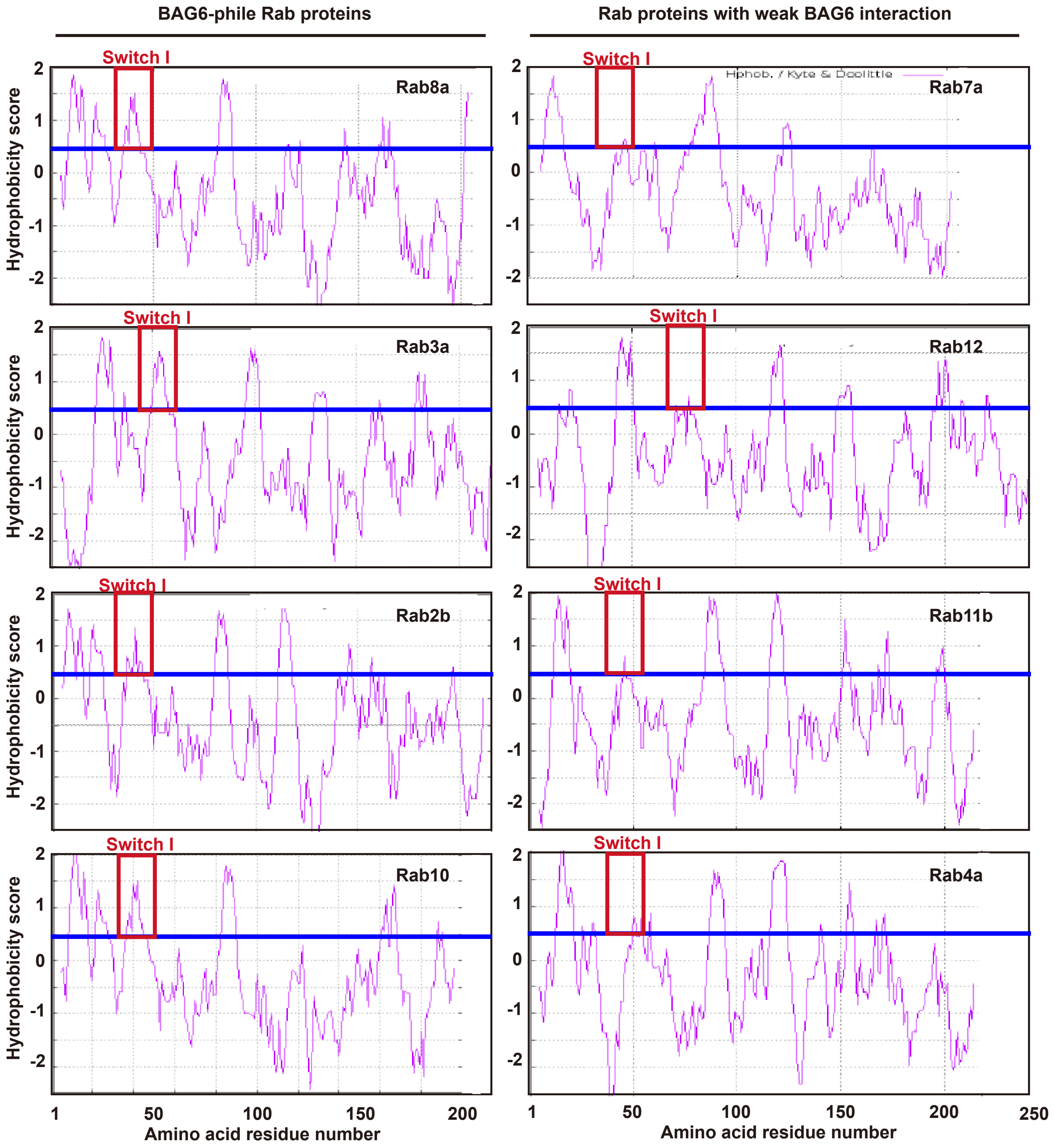

Appendix Figure S5, Takahashi et al

Appendix Figure S5.

Kyte-Doolittle hydrophobicity plots of BAG6-phile Rab proteins and that with weak BAG6 interaction (related to Appendix Fig S4).

The hydrophobicity peak within Switch I region of respective Rab proteins are indicated as red rectangle. The horizontal axis numbers denote the corresponding amino acid positions in these proteins.

Cell surface Lectin-Alexa Fluor 488 stain

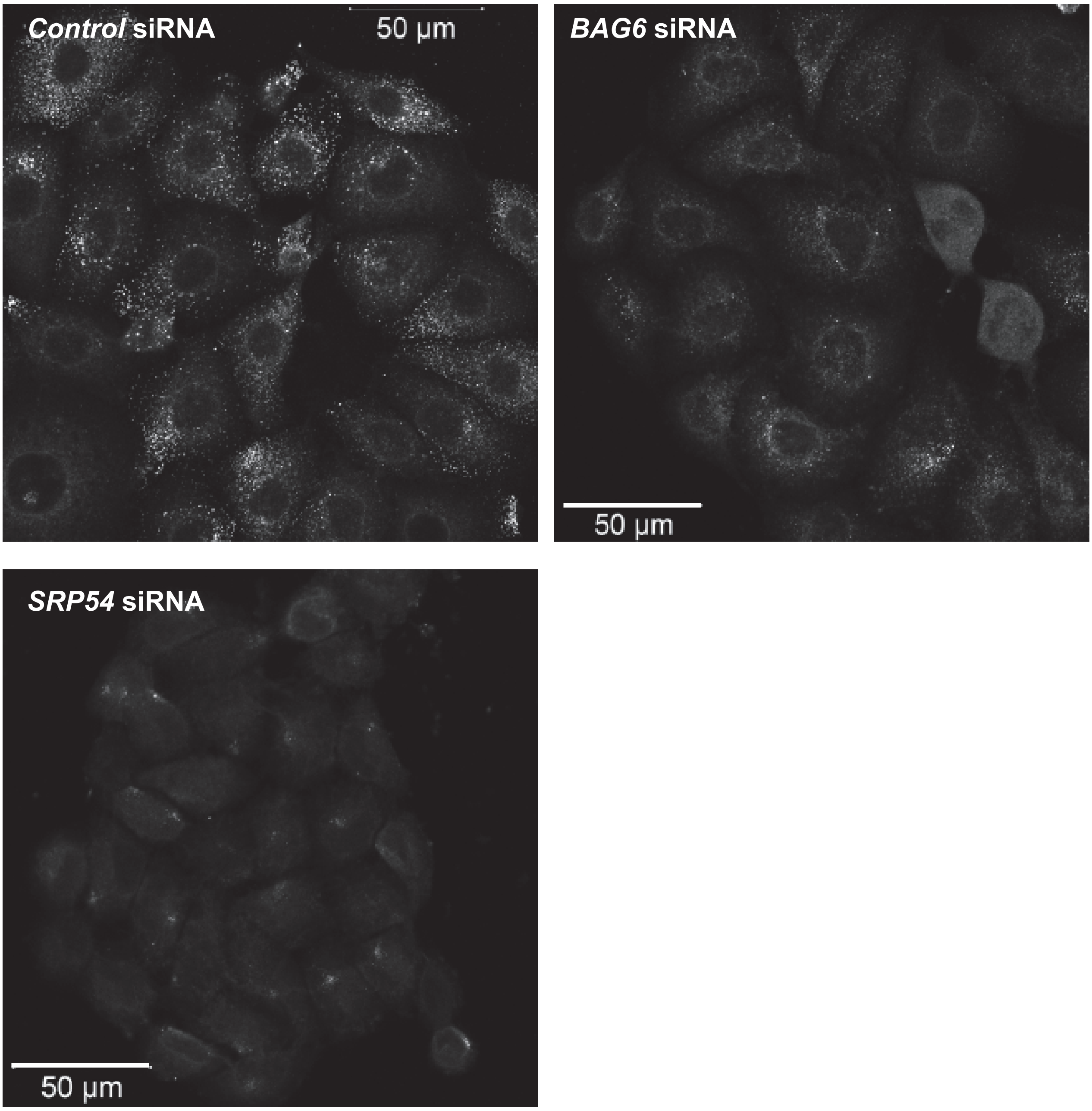

Appendix Figure S6, Takahashi et al

Appendix Figure S6.

The effects of BAG6 knockdown on the amount of cell surface glycoproteins (related to Fig 7).

Low magnification images from the cell surface glycoprotein quantification assay with Alexa Fluor^{TM}488-conjugated Lectin GS-II as a probe. Cell surface fluorescent signals were detected by laser scanning confocal microscopy without plasma membrane permeabilization as in Fig 7E. *BAG6* siRNA down-regulated the cell surface expression of glycoproteins. SRP54 knockdown was used as a positive control for this experiment and in the representative photograph used in Fig 7F. Scale bar: 50 μm.

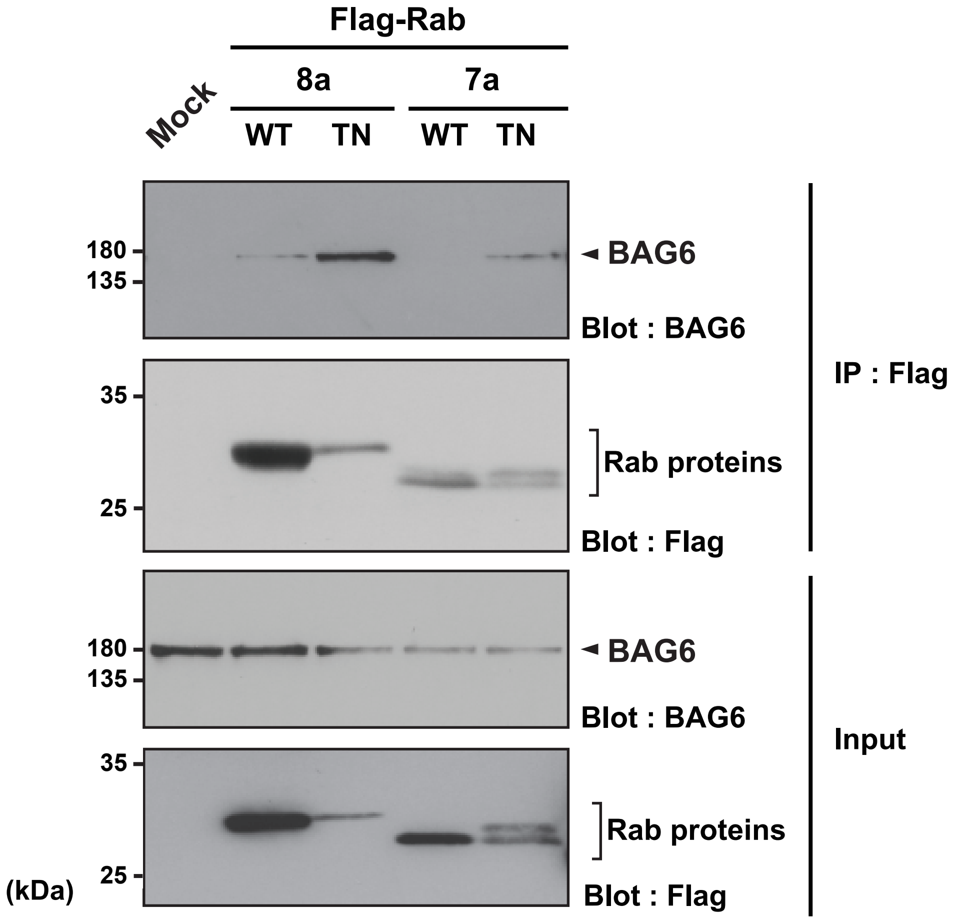

Appendix Figure S7, Takahashi et al

Appendix Figure S7.

Rab7a GDP mutant show increased affinity for BAG6 compared to the case in its wild type (related to Fig EV3). Flag-tagged WT Rab8a, WT Rab7a and their TN mutant derivatives (GDP-bound forms) were immunoprecipitated and probed with an anti-BAG6 antibody. MG-132 (10 μM) was included in the cell culture for 4 h.

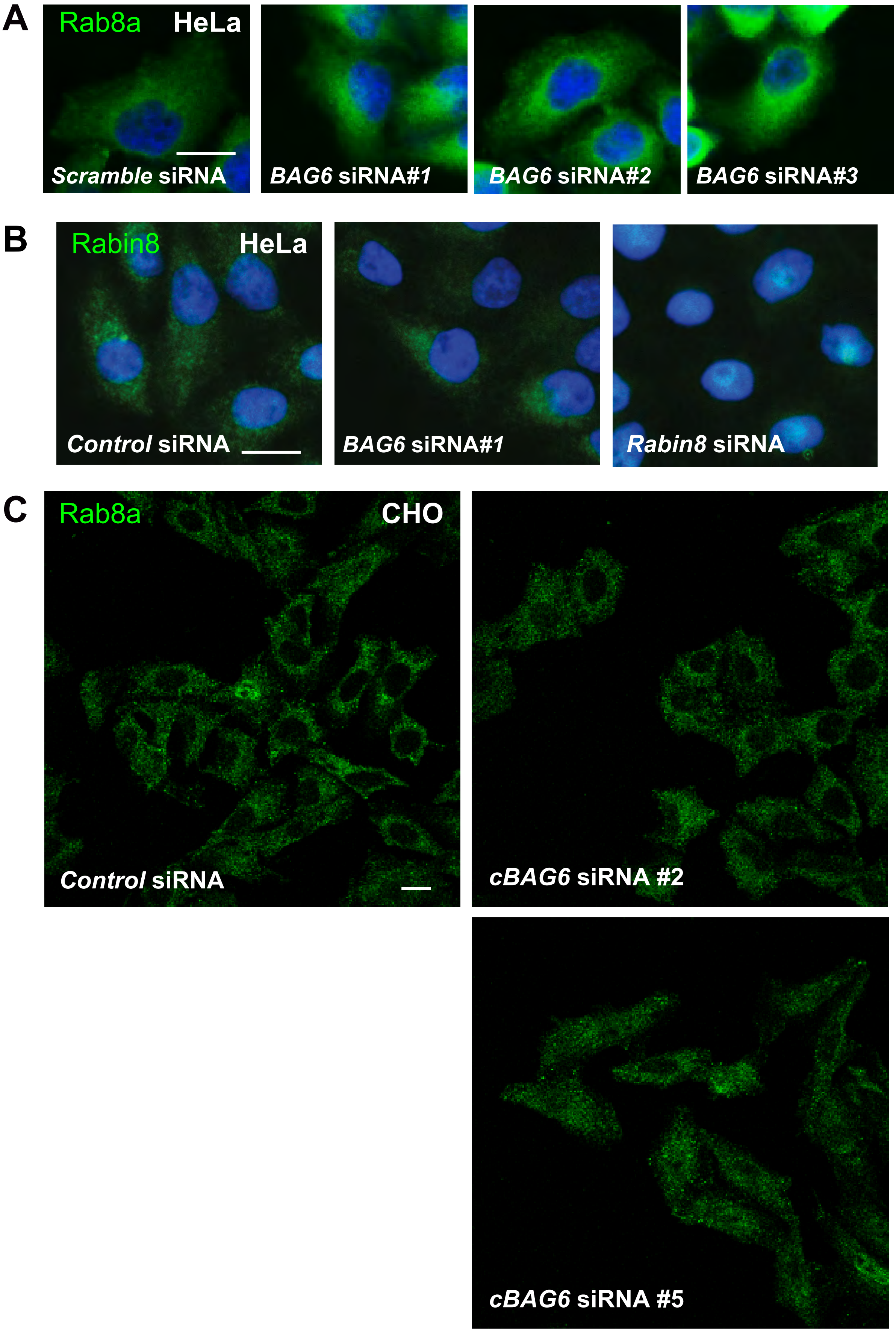

Appendix Figure S8, Takahashi et al

Appendix Figure S8.

No detectable abnormality of Rab8a and Rabin8 localizations in BAG6-suppressed cells (related to Fig EV4A).

Immunostaining of the endogenous Rab8a (A, C, shown as green) in HeLa (A) and CHO (C) cells. Immunostaining of the endogenous Rabin8 in HeLa cell (B, shown as green). The positions of the nucleus are shown by Hoechst staining (blue). All cells were transfected with a series of siRNA constructs as indicated. Scale bars: 10 μm.
