# Supplementary material for: Cytoplasmic control of Rab family small GTPases through BAG6
Source: EMBO Rep. 2019 Feb 25;20(4):e46794. doi: 10.15252/embr.201846794 (PMC6446207; doi:10.15252/embr.201846794)
Supplement: Supplementary file 2 — Expanded View Figures PDF [file EMBR-20-e46794-s002.pdf]

Expanded View Figures

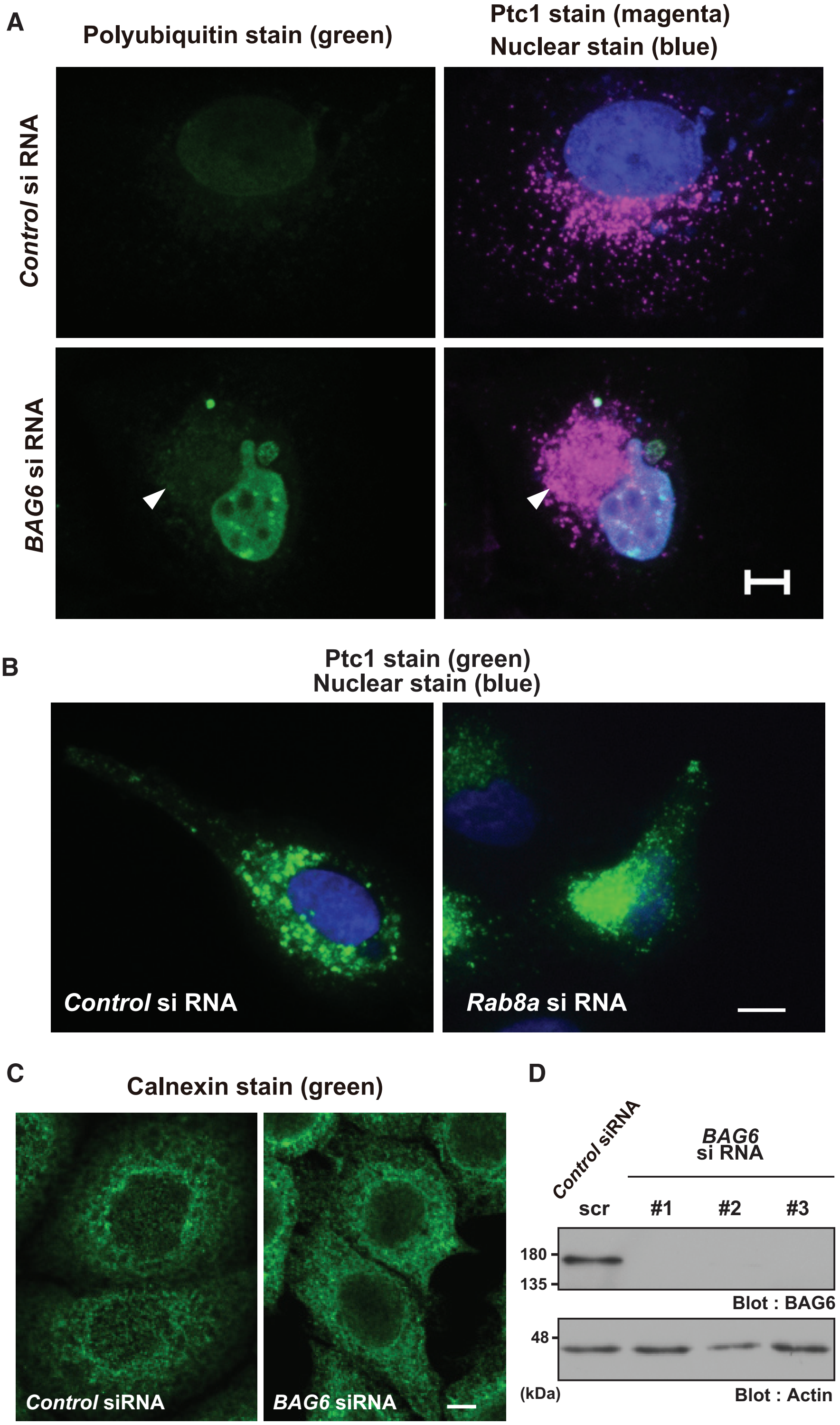

Figure EV1. BAG6 is essential for maintaining endosomal protein localization (related to Fig 1).

A The abnormal Ptc1 signal observed in BAG6-suppressed HeLa cells at the perinuclear region was not derived from protein aggregates. The Ptc1 immunosignal in BAG6-suppressed cells (shown as magenta with an arrowhead) was negative for FK2 polyubiquitin staining (shown as green), a marker for cytoplasmic protein aggregates. See also Appendix Fig S1A. Nuclei were stained by Hoechst 33342 (shown as blue). Scale bar: 10 μm.

B Immunostaining of Ptc1 (green) in HeLa cells that were treated with or without *Rab8a* siRNA. See also Appendix Fig S1D. Scale bar: 10 μm.

C Immunostaining of the ER luminal marker protein calnexin (green) in HeLa cells that were treated with or without *BAG6* siRNA. Scale bar: 5 μm.

D Cell lysates were subjected to Western blot analysis with an anti-BAG6 antibody to verify the knockdown efficacy of *BAG6* siRNA#1, #2, and #3 in HeLa cells. As a negative control, *BAG6* siRNA#1scr was used. Actin was used as a loading control.

Source data are available online for this figure.

© 2019 The Authors

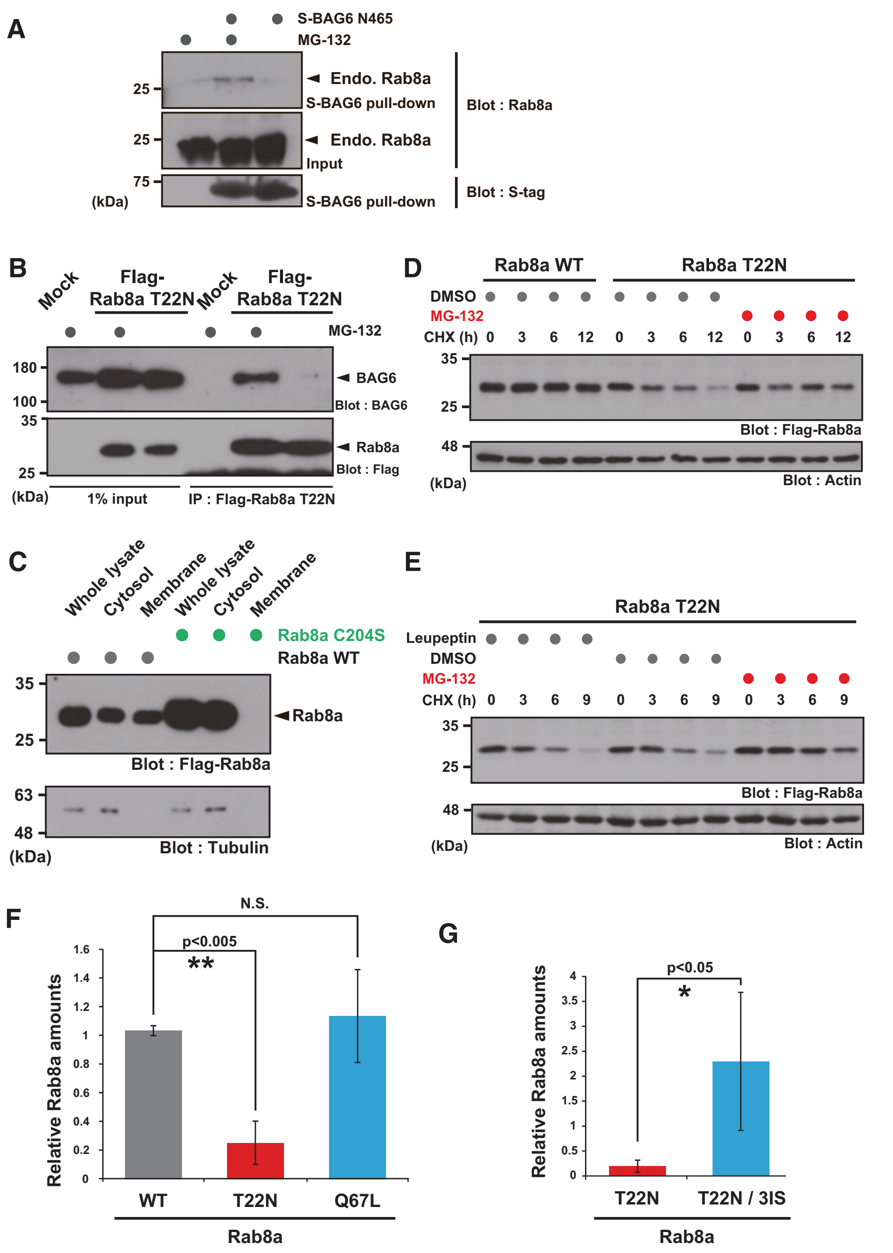

Figure EV2. The cytoplasmic inactive form of Rab8a is degraded by the proteasome pathway (related to Figs 2–4).

A Endogenous Rab8a protein was co-precipitated with S-tagged BAG6 N465 from HeLa cell lysate in the presence of MG-132.

B Co-immunoprecipitation of BAG6 with Rab8a was stimulated by the addition of a proteasome inhibitor. HeLa cells expressing Flag-tagged Rab8a (T22N) protein were treated with (+) or without (−) 10 μM MG-132. At 4 h after MG-132 treatment, the cells were lysed and Flag-precipitates were probed with an anti-BAG6 antibody.

C Rab8a C204S mutant protein localized exclusively in the soluble cytoplasmic fraction. Whole cell extracts of HeLa cells expressing Flag-tagged Rab8a proteins (WT or C204S mutant) were fractionated into the cytosolic and membrane fractions. Tubulin was used as a cytoplasmic marker.

D, E T22N mutant form of full-length Rab8a protein was stabilized by MG-132, while leupeptin did not affect its stability. At 24 h after Rab8a transfection, the cells were cultured with 10 μM MG-132, 10 μM leupeptin, or an equivalent amount of DMSO (as a negative control), and then chased with 20 μg/ml CHX and harvested at the indicated times after CHX addition.

F, G Anti-Flag immunosignals of Rab8a WT, T22N, Q67L, and T22N-3IS proteins in Figs 3E and 4D were quantified. The data represent the mean ± SD calculated from three independent biological replicates (n = 3). *$P < 0.05$ compared with control siRNA. N.S. indicates not significant (Student's t-test).

Source data are available online for this figure.

　© 2019 The Authors

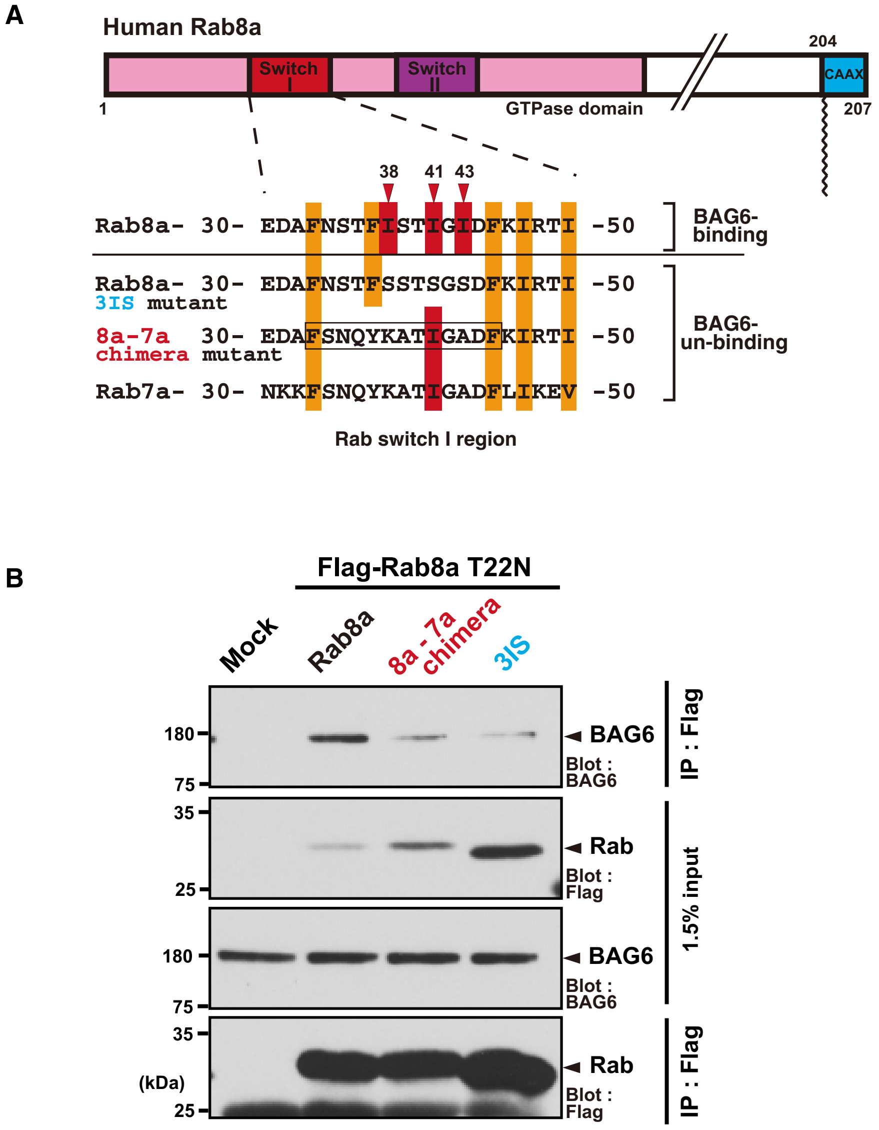

Figure EV3. The Switch I region of Rab8a is critical for its interaction with BAG6 (related to Fig 4).

A Schematic representation of the Switch I region of the Rab8a-Rab7a chimeric protein. The amino acid residues 33–45 (boxed region) of full-length Rab8a were substituted with those of Rab7a and this mutant protein was designated as the “8a-7a chimera”. The numbers denote the corresponding amino acids of human Rab8a and Rab7a.

B The 8a-7a chimera protein with the T22N mutation showed greatly reduced affinity with BAG6 compared with the case in Rab8a (T22N).

Source data are available online for this figure.

© 2019 The Authors

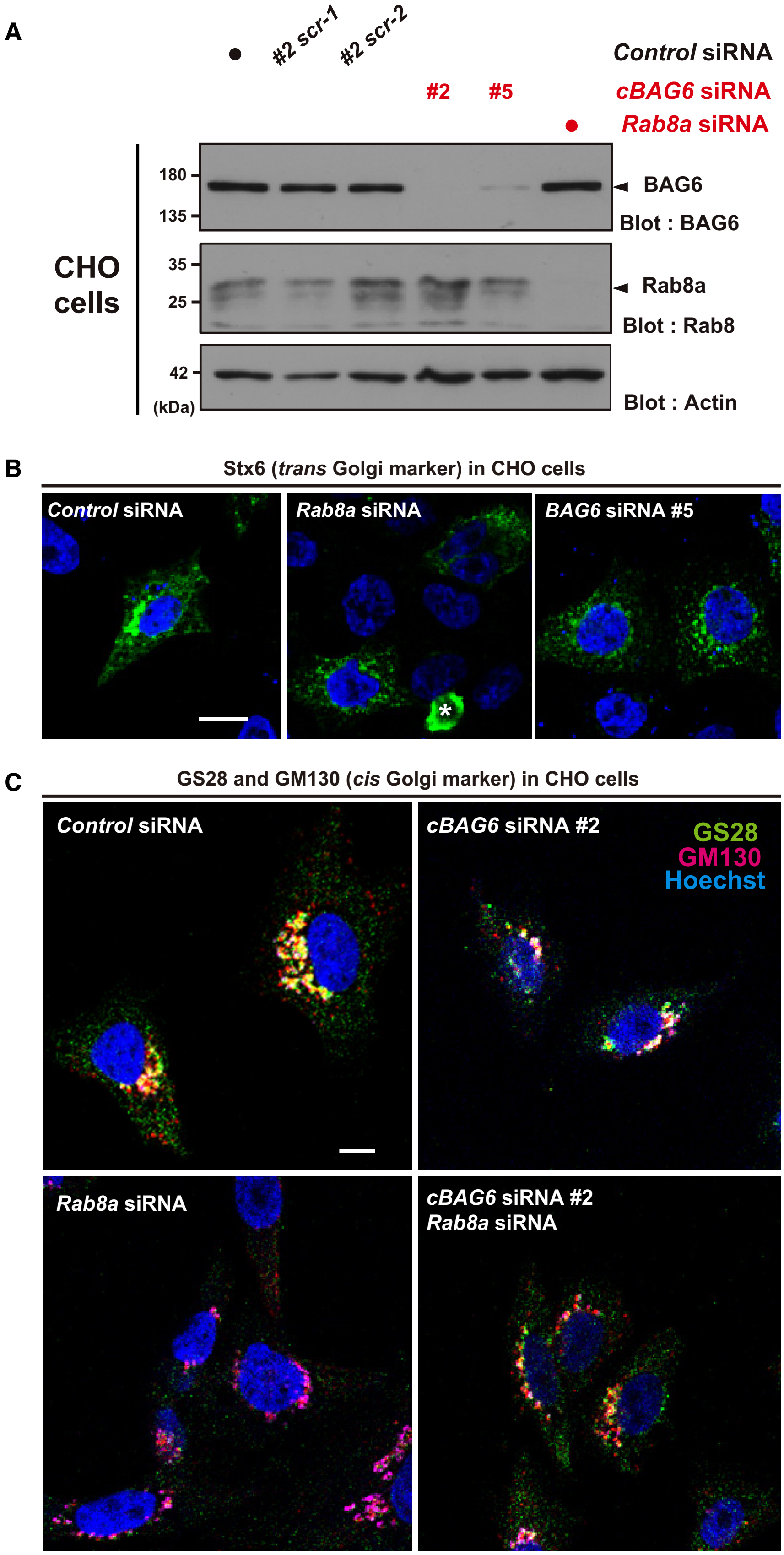

Figure EV4. The efficacy of BAG6 and Rab8a knockdown in CHO cells was examined (related to Fig 7).

A Verification of the depletion efficacy and specificity of two independent double-stranded RNAs (*cBAG6* siRNA#2 and #5) in rodent CHO cells. Note that the target sequences of these rodent *cBAG6* siRNAs are completely different to those of the human siRNAs used in Fig 1 (see Materials and Methods). Two scrambled sequences for *cBAG6* siRNA#2 (designated #2scr-1 and #2scr-2, respectively), as well as MISSION siRNA Universal Negative Control 1 (indicated by a black dot), were used as negative controls. Efficacy of Rab8a knockdown in CHO cells was also verified by a blot with endogenous proteins.

B Comparison of the defects observed in the distribution of Stx6 (green) with siRNAs (*cBAG6* siRNA#5 and *Rab8a* siRNA#1, respectively) in CHO cells. Note that both *cBAG6* siRNA#5 and #2 are Chinese hamster-specific, while the target sequence of *Rab8a* siRNA#1 is identical between humans and hamsters. An asterisk indicates a non-specific signal. Scale bar: 10 μm.

C Comparison of the defects observed in the distribution of the ER-Golgi SNARE protein GS28 (green) and the *cis*-Golgi marker GM130 (red) with siRNAs (*cBAG6* siRNA#2 or *RAB8a* siRNA#1 and their combination) in CHO cells. GS28 and GM130 signals were dispersed throughout the perinuclear region of the cytoplasm in Rab8a knockdown cells, a similar phenotype to that observed in BAG6-depleted cells. Scale bar: 10 μm.

Source data are available online for this figure.

© 2019 The Authors

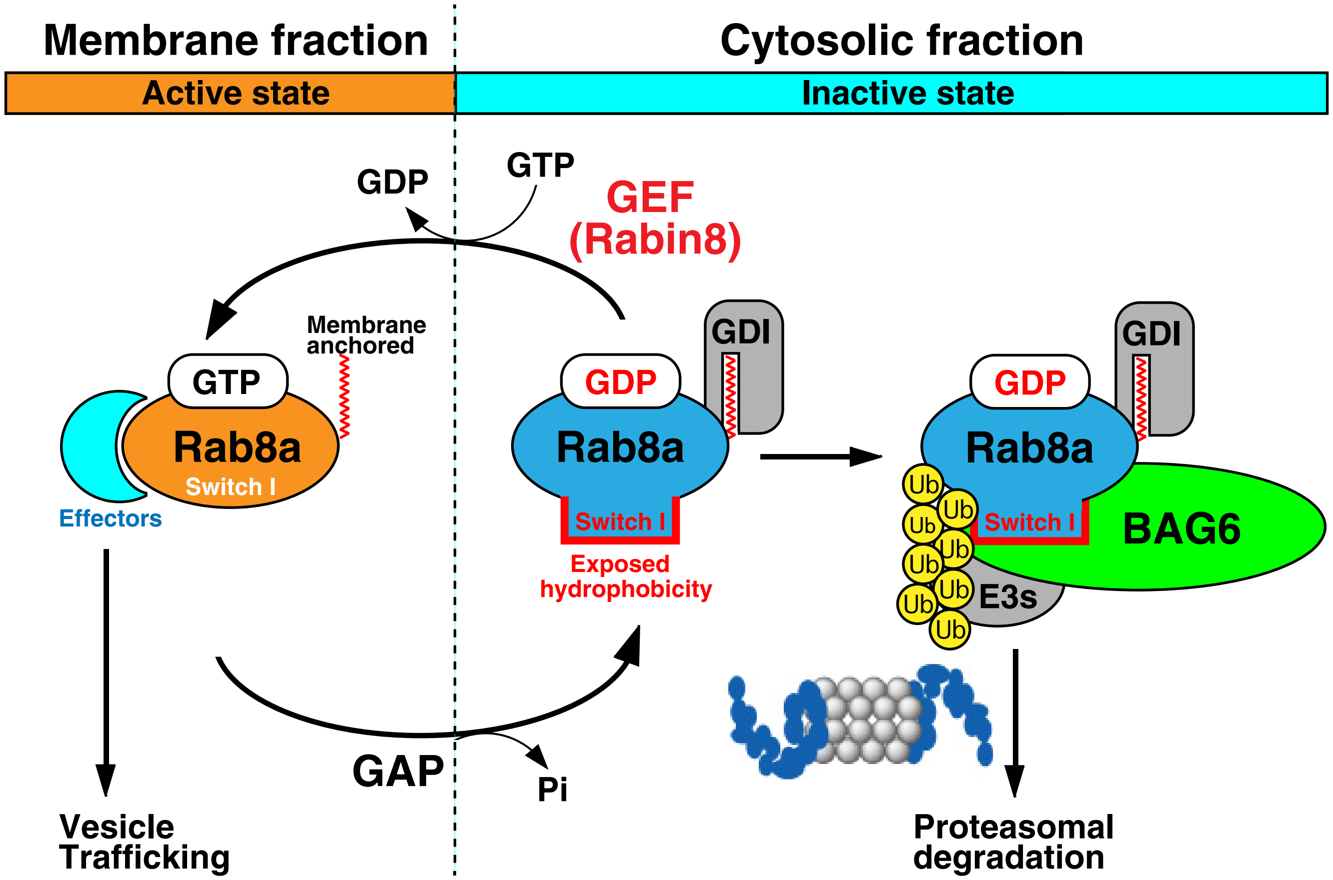

Figure EV5. BAG6 targets the GDP-associated cytoplasmic form of Rab8a for degradation.
The folding of Rab family GTPases is dependent on the bound nucleotide, and Rab8a (GDP-bound form) shows remarkable instability *in vivo*. The exposed hydrophobicity of the Rab8a Switch I region (GDP-bound form) is essential for its ubiquitin-mediated degradation, and thus prevent the excess accumulation of inactive Rab species during the course of GDP-GTP cycling.
