# Supplementary figures and images for: Cytoplasmic control of Rab family small GTPases through BAG6
Source: EMBO Rep. 2019 Feb 25;20(4):e46794. doi: 10.15252/embr.201846794 (PMC6446207; doi:10.15252/embr.201846794)

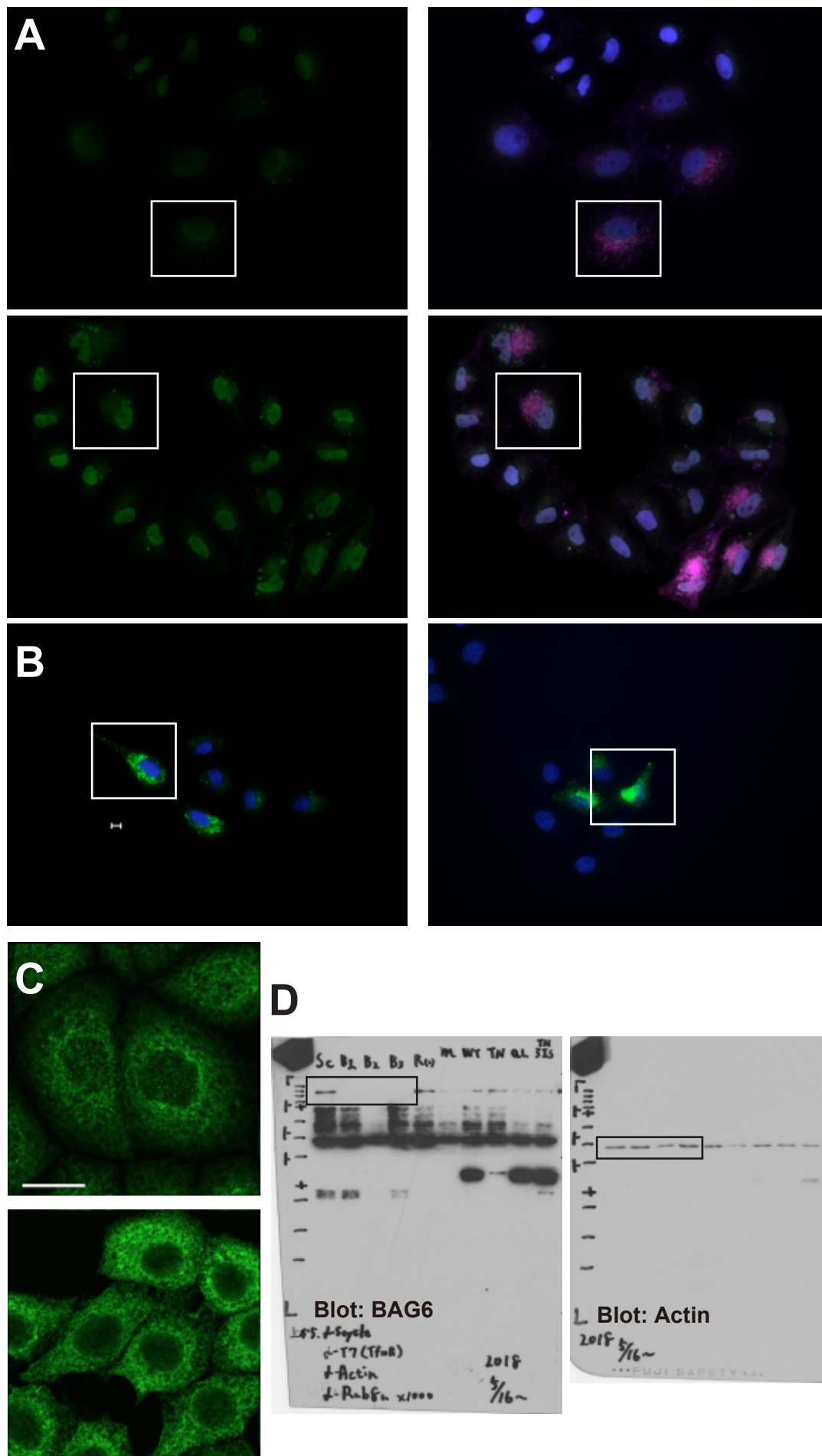

Figure EV1, Takahashi et al  
Original Source Data

Supplement: Supplementary file 3 — Source Data for Expanded View [file EMBR-20-e46794-s003.zip › Source_Data_for_EV_Figures/Source_Data_for_FigureEV1.pdf]

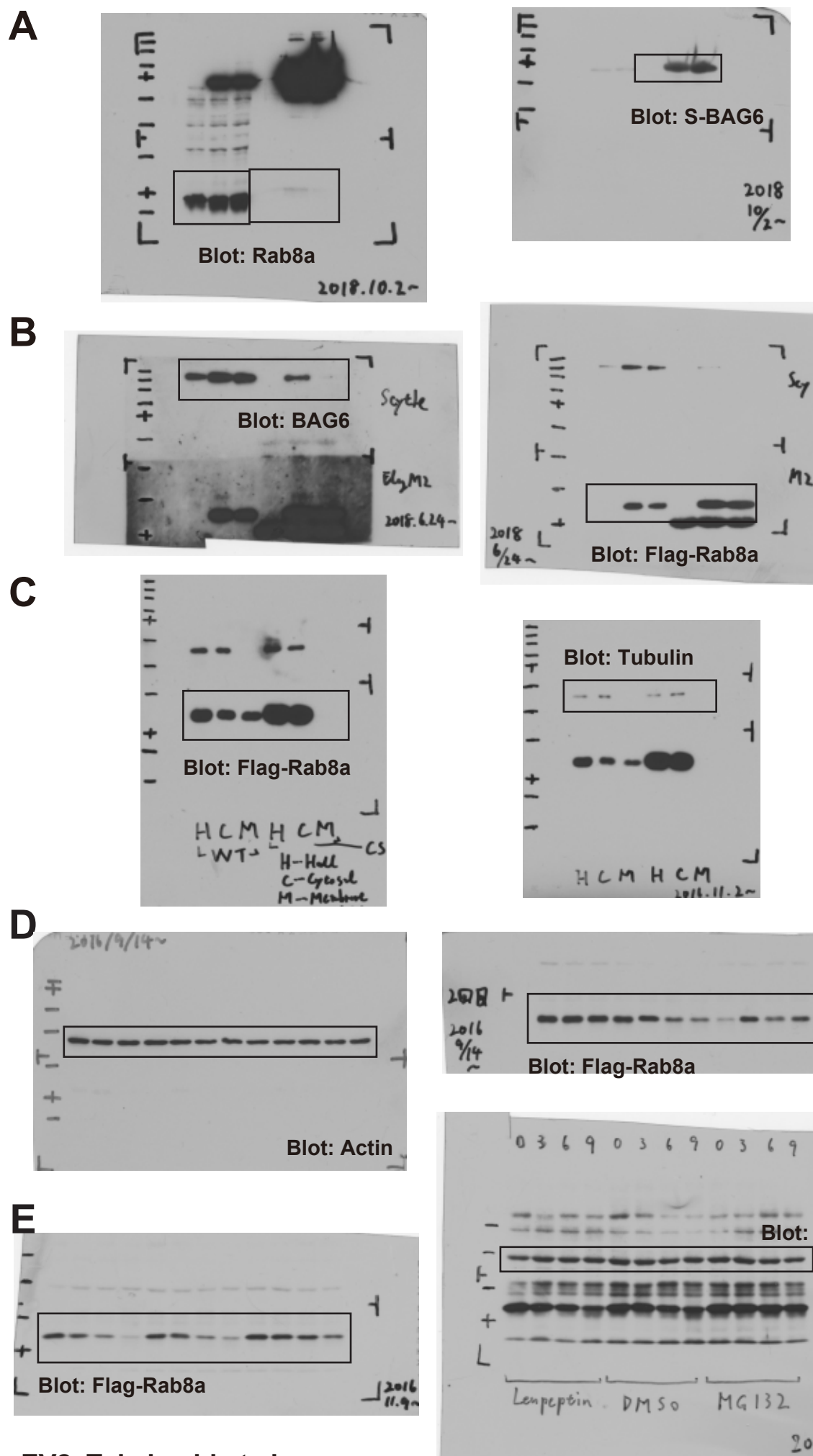

Figure EV2, Takahashi et al  
Original Source Data

Supplement: Supplementary file 3 — Source Data for Expanded View [file EMBR-20-e46794-s003.zip › Source_Data_for_EV_Figures/Source_Data_for_FigureEV2.pdf]

**B**

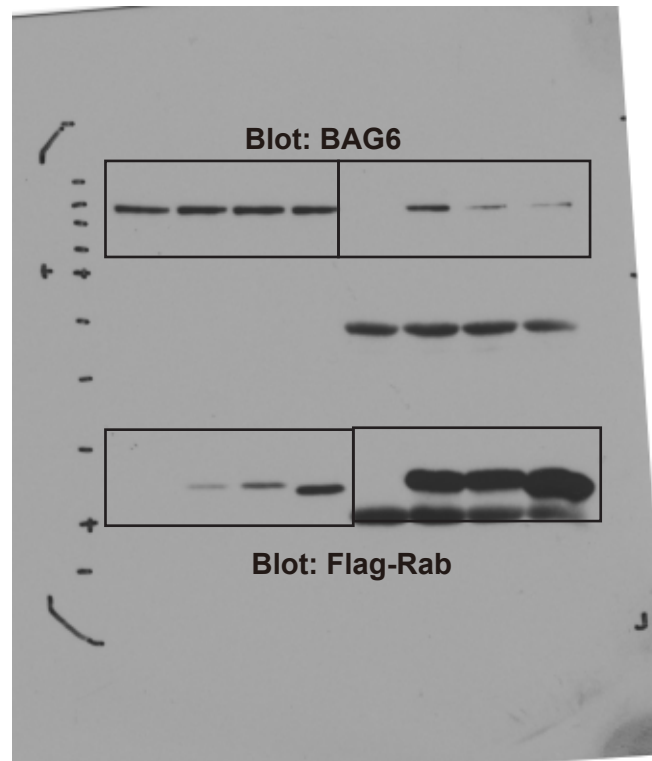

Figure EV3, Takahashi et al  
Original Source Data

Supplement: Supplementary file 3 — Source Data for Expanded View [file EMBR-20-e46794-s003.zip › Source_Data_for_EV_Figures/Source_Data_for_FigureEV3.pdf]

**A**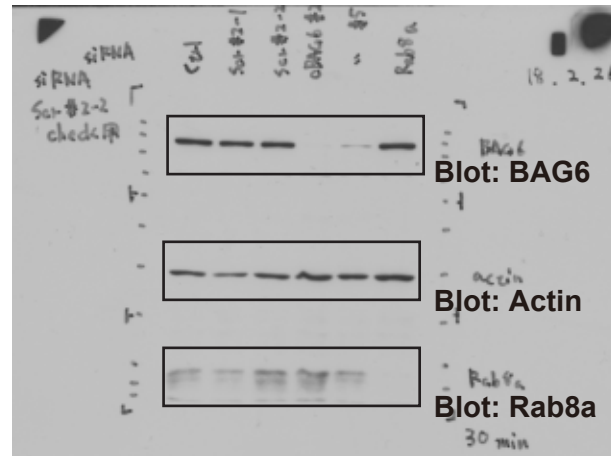**B**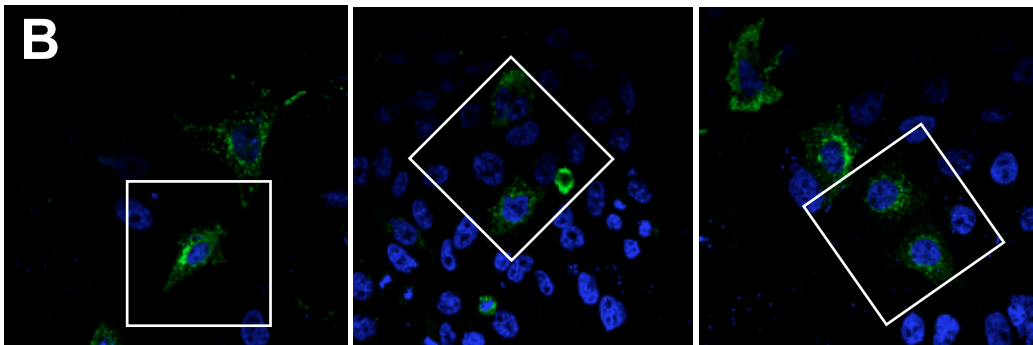**C**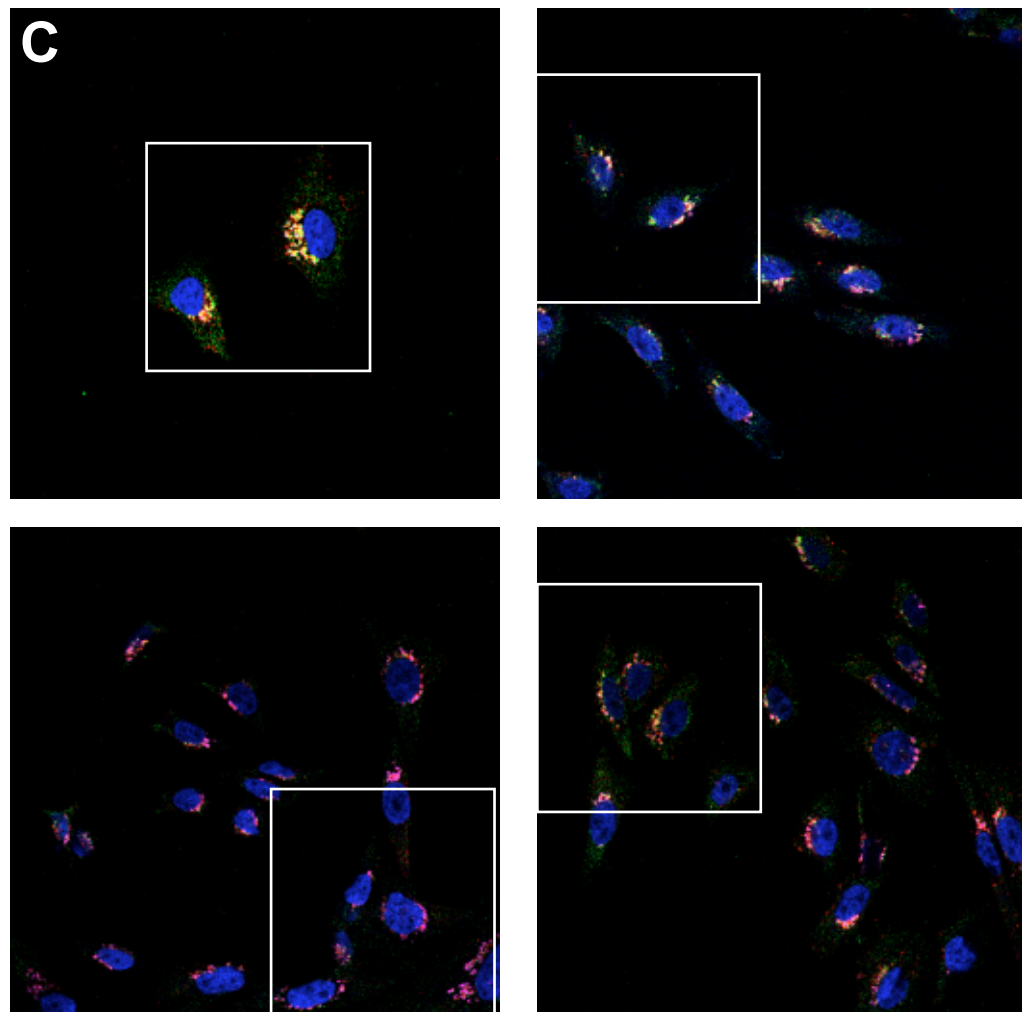

Figure EV4, Takahashi et al  
Original Source Data

Supplement: Supplementary file 3 — Source Data for Expanded View [file EMBR-20-e46794-s003.zip › Source_Data_for_EV_Figures/Source_Data_for_FigureEV4.pdf]

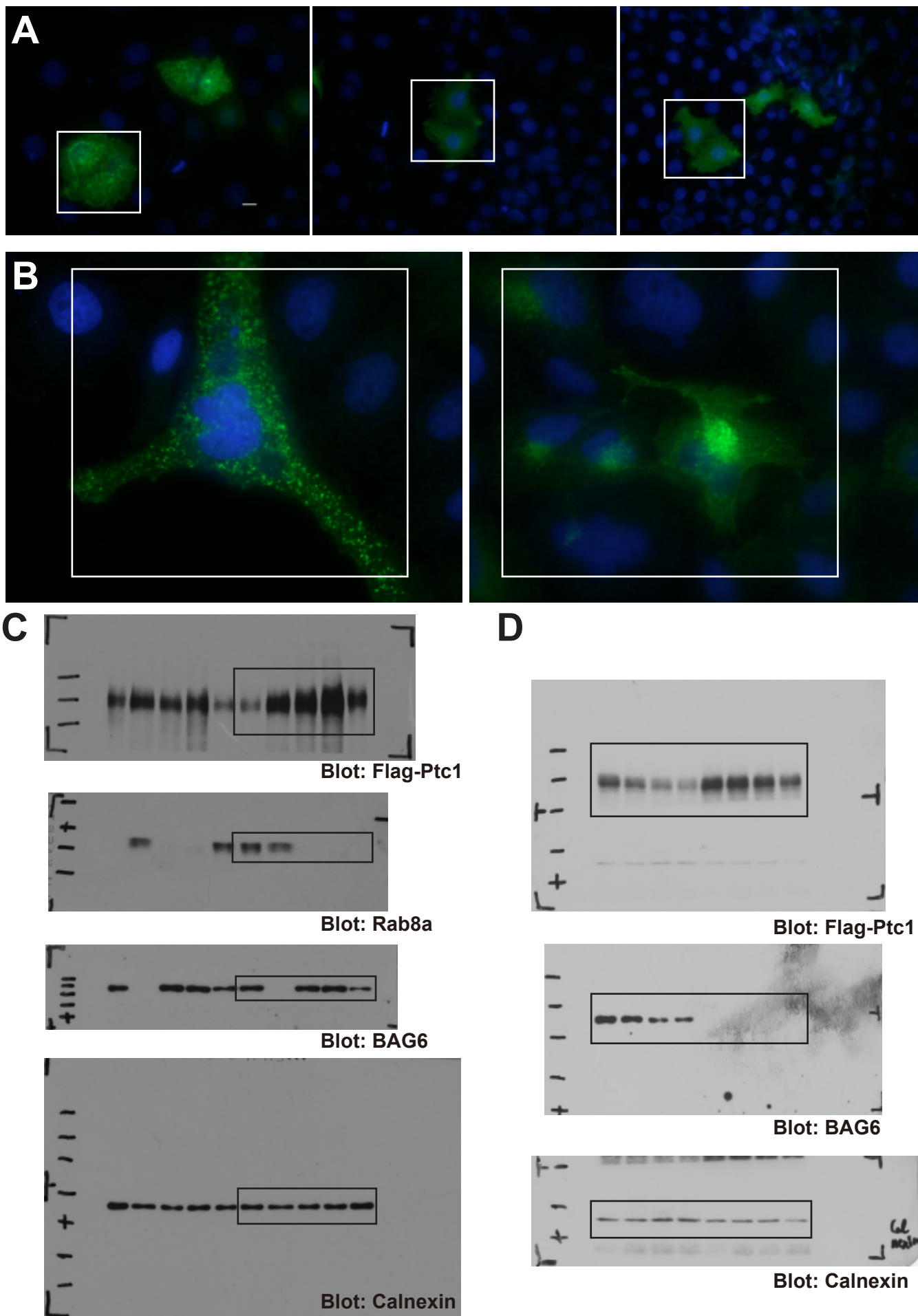

Figure 1, Takahashi et al  
Original Source Data

Supplement: Supplementary file 5 — Source Data for Figure 1 [file EMBR-20-e46794-s004.pdf]

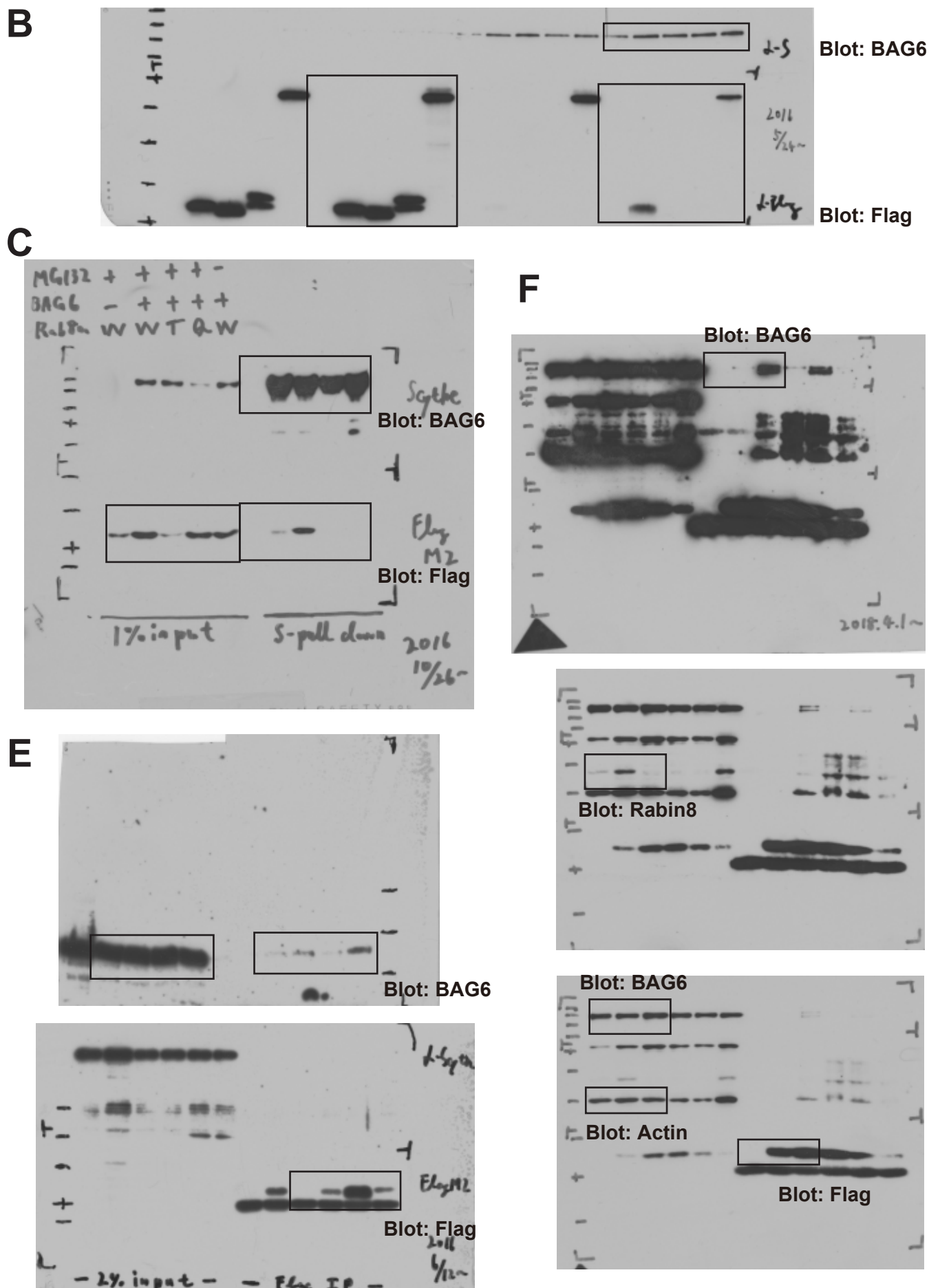

Figure 2, Takahashi et al  
Original Source Data

Supplement: Supplementary file 6 — Source Data for Figure 2 [file EMBR-20-e46794-s005.pdf]

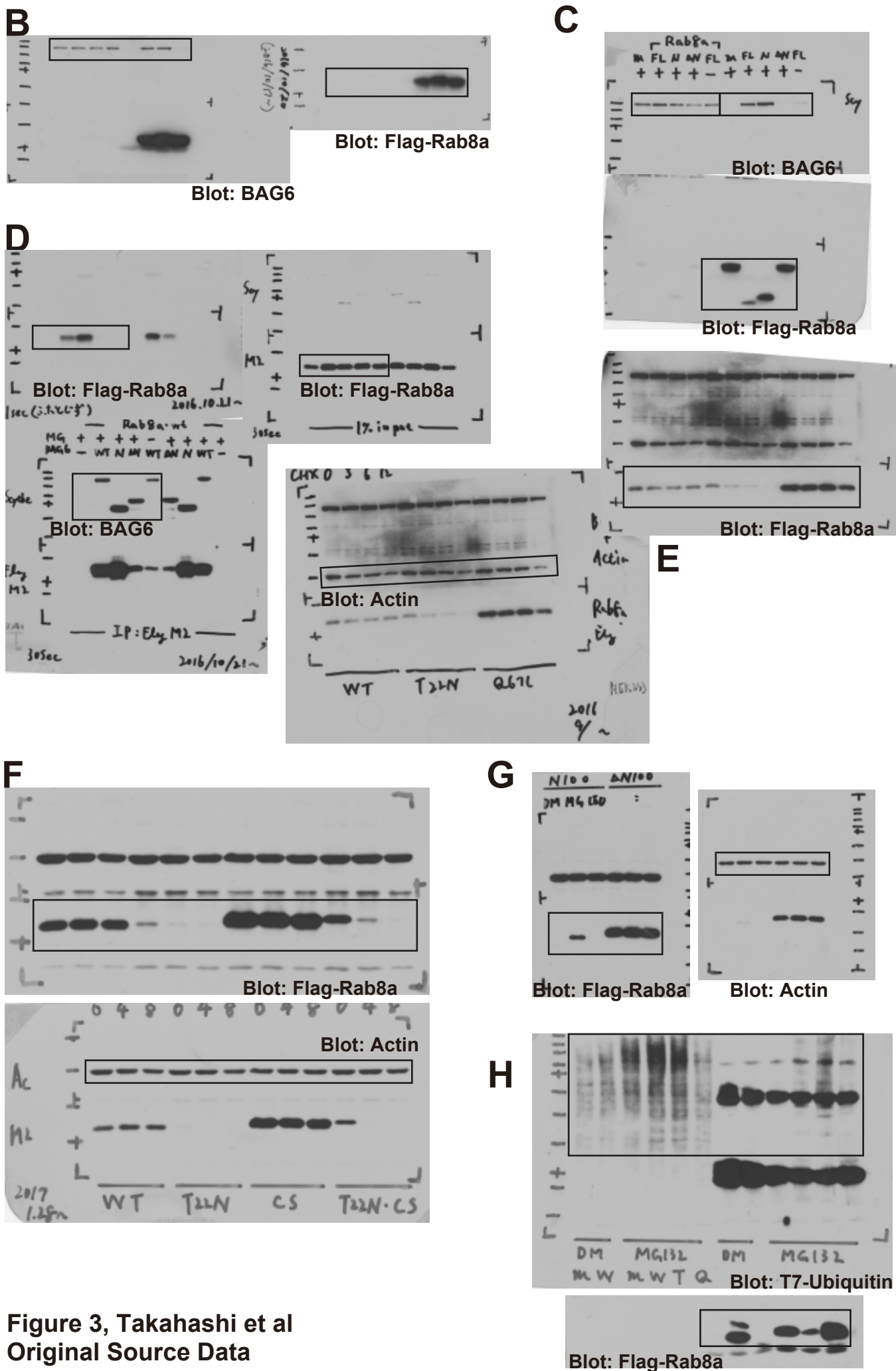

Figure 3, Takahashi et al  
Original Source Data

Supplement: Supplementary file 7 — Source Data for Figure 3 [file EMBR-20-e46794-s006.pdf]

**C**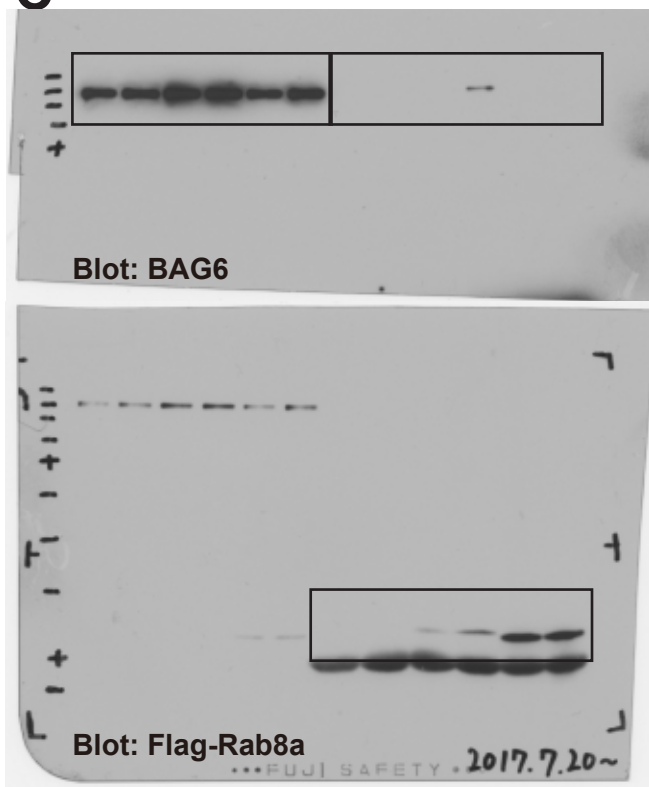**D**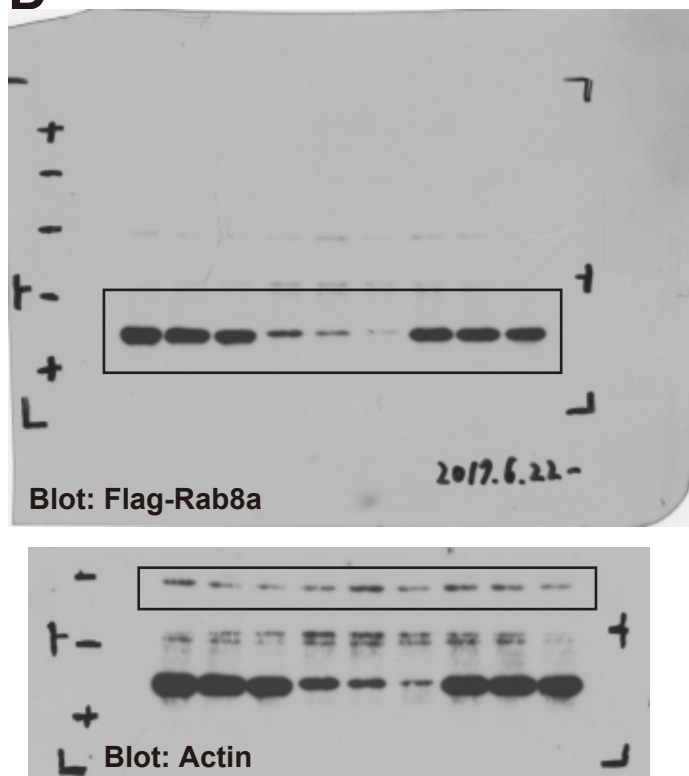**E**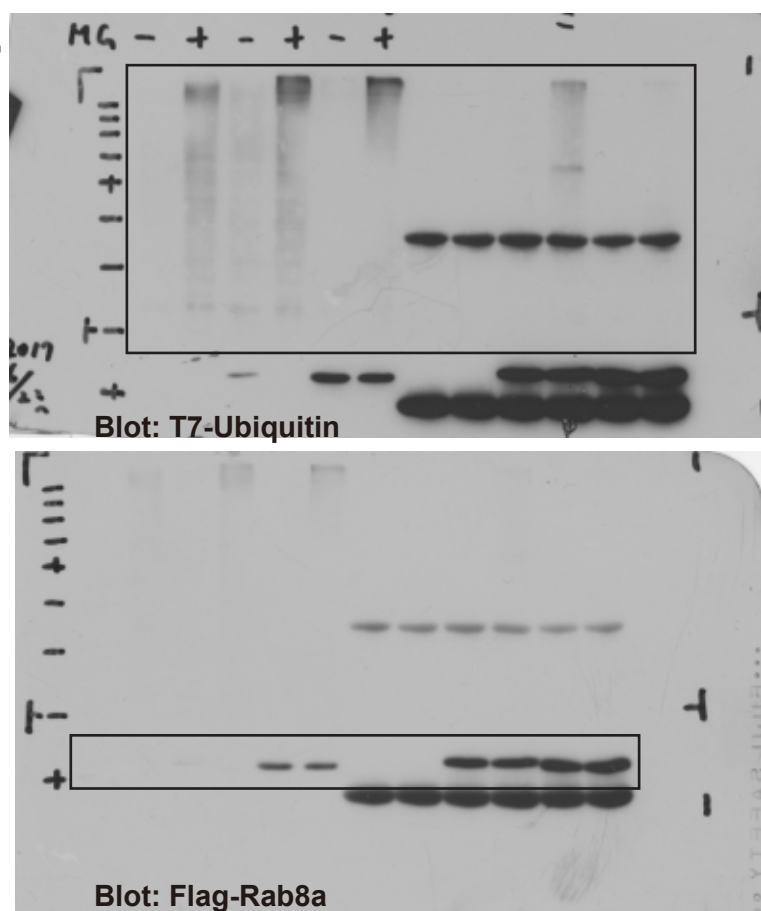

Figure 4, Takahashi et al  
Original Source Data

Supplement: Supplementary file 8 — Source Data for Figure 4 [file EMBR-20-e46794-s007.pdf]

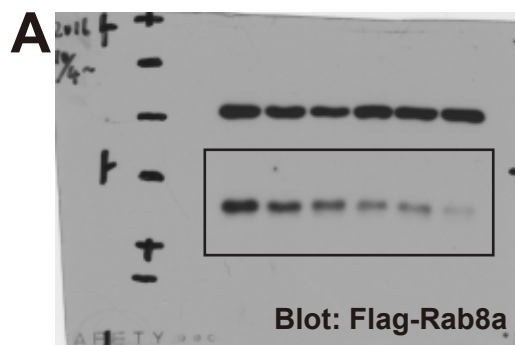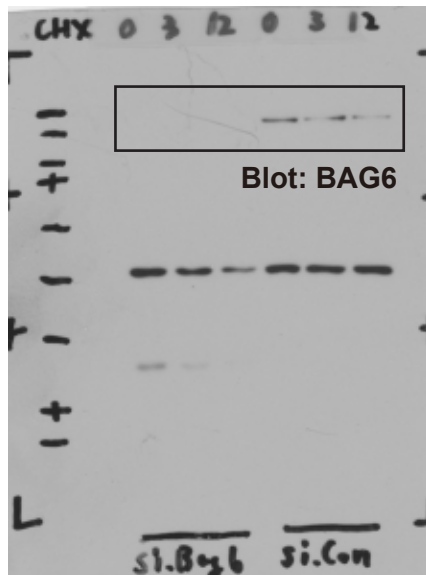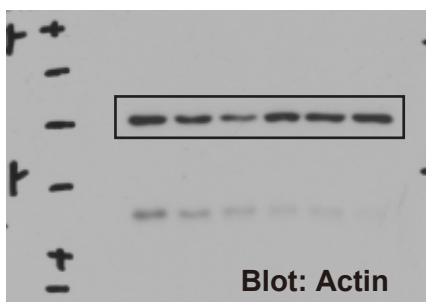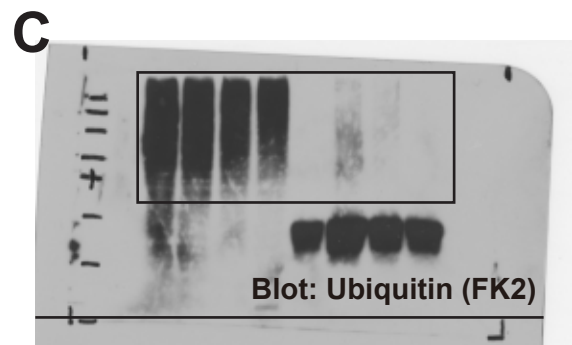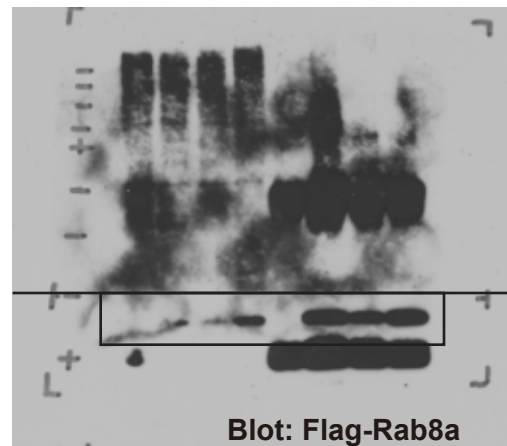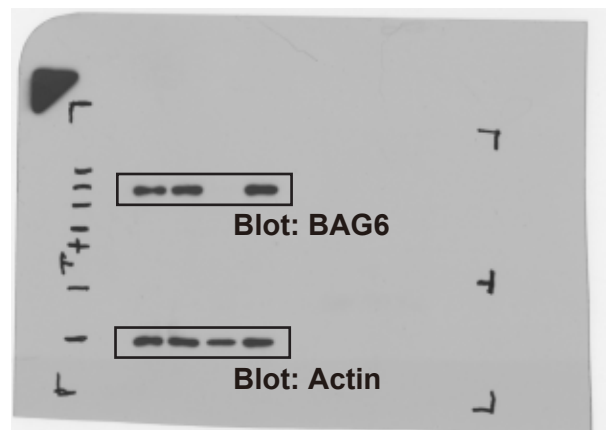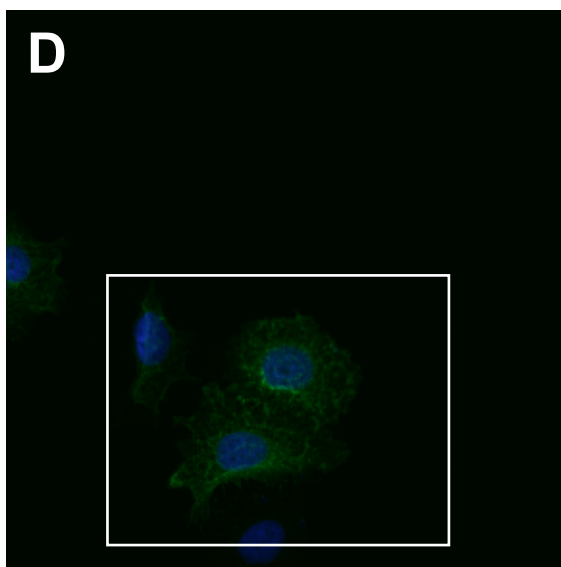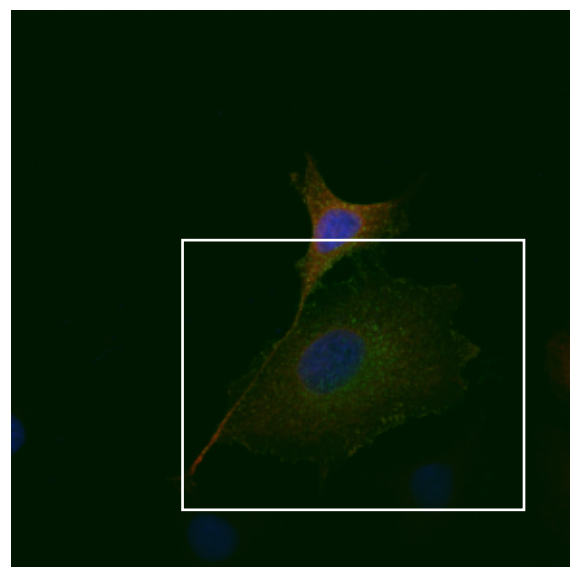

Figure 5, Takahashi et al  
Original Source Data

Supplement: Supplementary file 9 — Source Data for Figure 5 [file EMBR-20-e46794-s008.pdf]

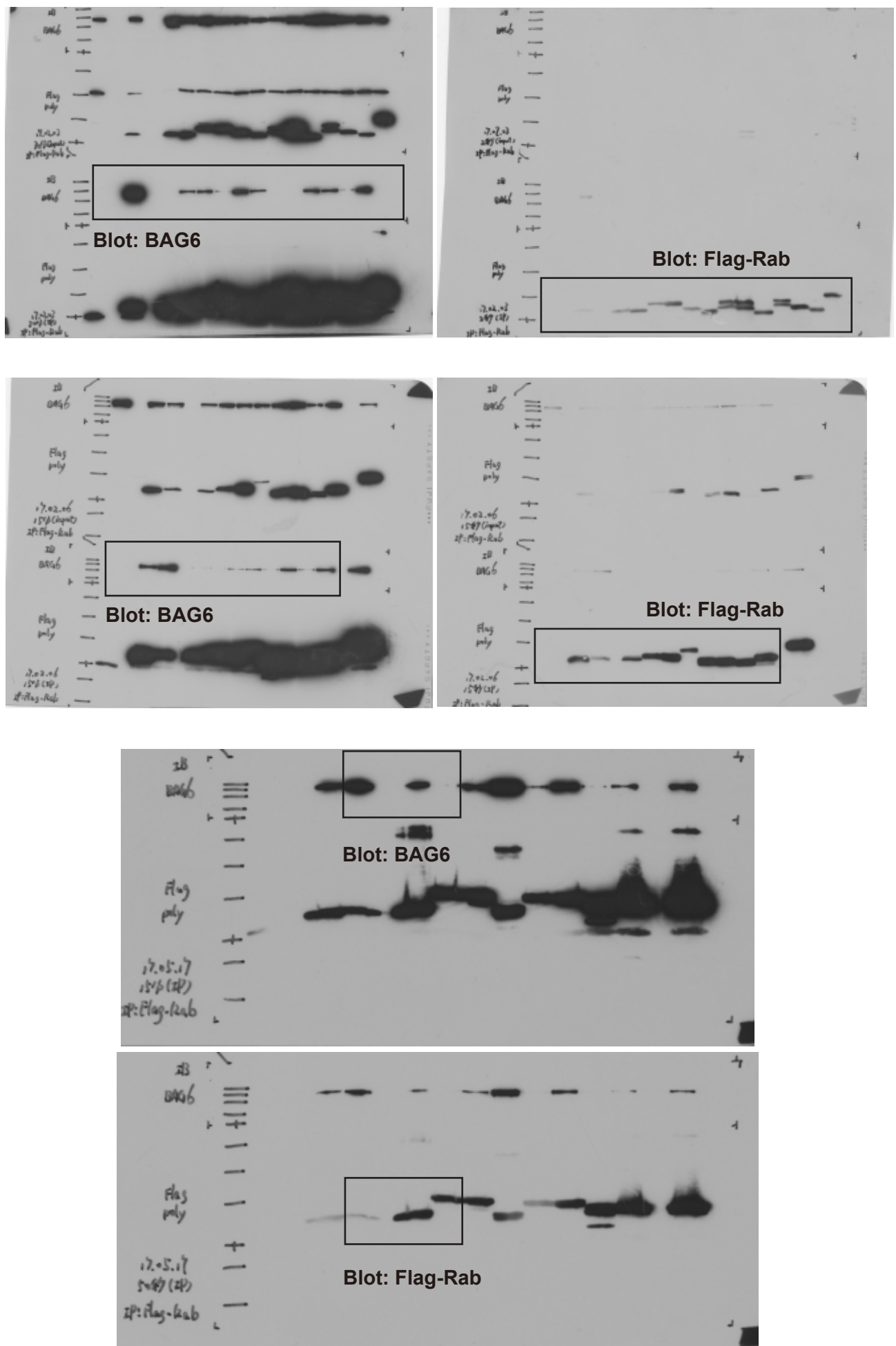

**Figure 6, Takahashi et al**  
Original Source Data

Supplement: Supplementary file 10 — Source Data for Figure 6 [file EMBR-20-e46794-s009.pdf]

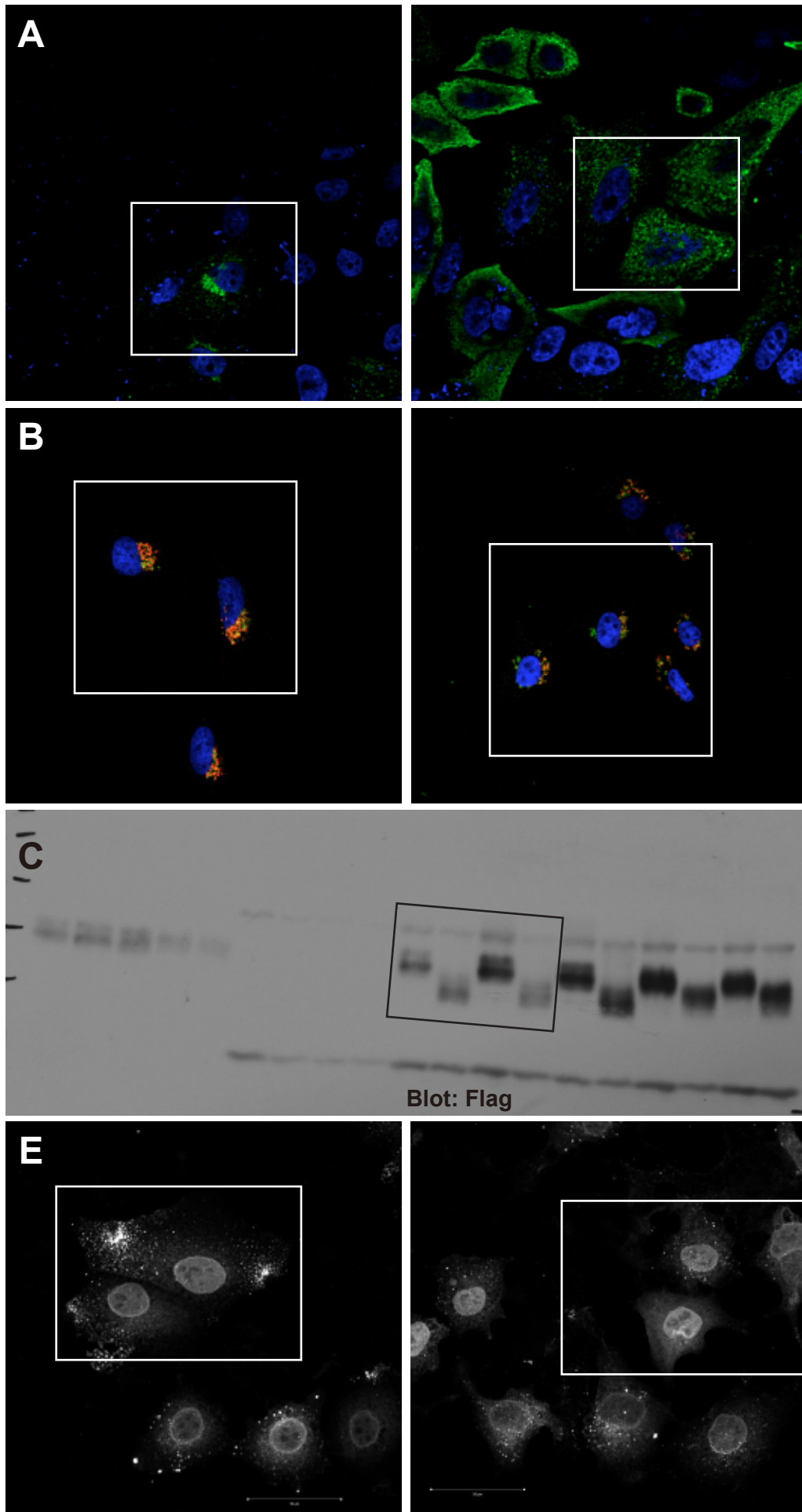

Figure 7, Takahashi et al  
Original Source Data

Supplement: Supplementary file 11 — Source Data for Figure 7 [file EMBR-20-e46794-s010.pdf]
